# Valorization of *Boehmeria nivea* stalk towards multipurpose fractionation: furfural, pulp, and phenolic monomers

**DOI:** 10.1186/s13068-023-02351-x

**Published:** 2023-06-12

**Authors:** Zhen Zhang, Furong Tao, Hairui Ji

**Affiliations:** 1 Key Laboratory of Pulp and Paper Science and Technology of Ministry of Education, State Key Laboratory of Biobased Material and Green Papermaking, Faculty of Light Industry, Qilu University of Technology (Shandong Academy of Sciences), Jinan, 250353 China; 2 Faculty of Chemistry and Chemical Engineering, Qilu University of Technology (Shandong Academy of Sciences), Jinan, 250353 China

**Keywords:** *Boehmeria nivea* stalk valorization, Furfural, Papermaking, Phenolic monomers

## Abstract

**Background:**

As one of the most abundant bioresource in nature, the value-added utilization of lignocellulosic biomass is limited due to its inherent stubbornness. Pretreatment is a necessary step to break down the recalcitrance of cell walls and achieve an efficient separation of three main components (cellulose, hemicelluloses, and lignin).

**Results:**

In this study, hemicelluloses and lignin in *Boehmeria nivea* stalks were selectively extracted with a recyclable acid hydrotrope, an aqueous solution of *P*-toluenesulfonic acid (*p*-TsOH). 79.86% of hemicelluloses and 90.24% of lignin were removed under a mild pretreatment condition, C80T80t20, (acid concentration of 80 wt%, pretreatment temperature and time of 80 °C and 20 min, respectively). After ultrasonic treatment for 10 s, the residual cellulose-rich solid was directly converted into pulp. Subsequently, the latter was utilized to produce paper via mixing with softwood pulp. The prepared handsheets with a pulp addition of 15 wt% displayed higher tear strength (8.31 mN m^2^/g) and tensile strength (8.03 Nm/g) than that of pure softwood pulp. What’s more, the hydrolysates of hemicelluloses and the extracted lignin were transformed to furfural and phenolic monomers with yields of 54.67% and 65.3%, respectively.

**Conclusions:**

The lignocellulosic biomass, *Boehmeria nivea* stalks, were valorized to pulp, furfural, and phenolic monomers, successfully. And a potential solution of comprehensive utilization of *Boehmeria nivea* stalks was provided in this paper.

**Graphical Abstract:**

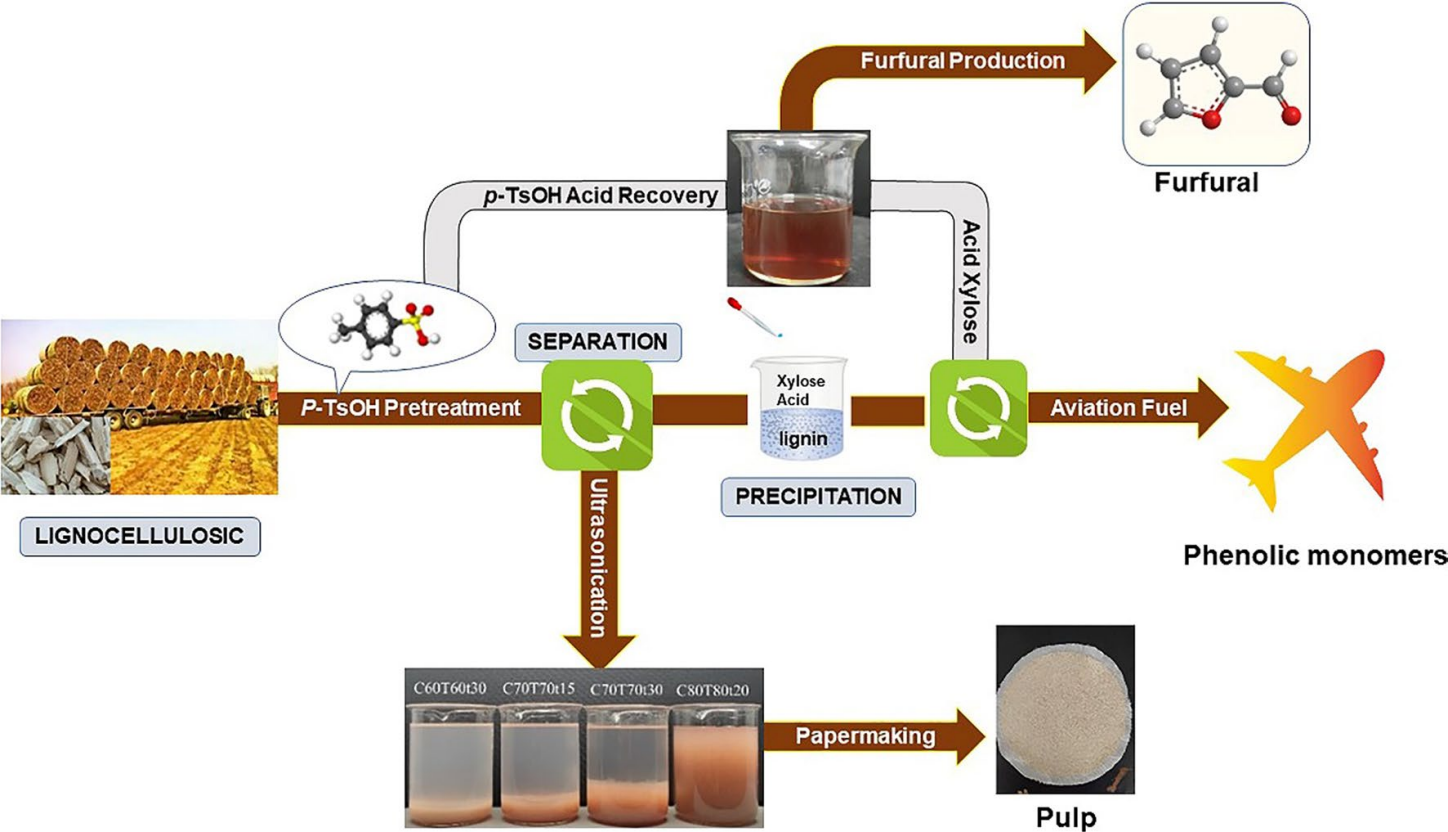

**Supplementary Information:**

The online version contains supplementary material available at 10.1186/s13068-023-02351-x.

## Background

Lignocellulosic biomass is one of the most abundant forms of terrestrial bioresource in nature. It mainly contains three biopolymers, namely, cellulose, hemicelluloses, and lignin [1]. In plant cell wall, lignin surrounds hemicelluloses and cellulose, while cellulose fibers are interlaced with hemicelluloses. This complex matrix provides cell wall with sufficient mechanical strength and rigidity to resist biodegradation [2, 3]. However, the inherently recalcitrant also makes lignocellulosic biomass more difficult to be used during its fractionation and conversion [4, 5]. Therefore, removing lignin in cell wall and destroying the natural recalcitrance are very important for the efficient separation of main constituents and subsequent utilization [6, 7].

In pulping and papermaking industry, the dissolution of lignin is mainly through cooking biomass in an aqueous mixture of sodium hydroxide and sodium sulfide (Kraft process) or in aqueous magnesium bisulfite solution (Sulfite process) [8, 9]. During extraction procedure, lignin usually undergoes some side reactions, for example, the dehydration, condensation, and uncontrolled repolymerization, resulting in the formation of recalcitrant carbon–carbon bonds, which brought many challenges in its upgrading and transforming utilization [10]. At the same time, a high temperature of 160–180 degree Celsius was employed during cooking resulting in high energy consumption [11]. In fact, lignin contains many functional groups, such as benzene rings, hydroxy, and carboxyl groups. Similarly, it has shown huge potential in the applications of bio-jet fuels, biodegradable polymers, antioxidants, dispersants, UV-absorbents, and phenolic resins [12]. Therefore, lignin is one of the most promising renewable sources of aromatics. However, the chemical structure destruction of lignin caused a reduction of its application value [13]. Thus, an effective pretreatment approach needs to be explored, necessarily, which can not only efficiently remove lignin in lignocellulosic biomass, but also retain its application value. Based on this, several technologies of dissolving lignin were developed in recent years. For example, some organic solvents concluding ethanol, acetone, acetic acid, oxalic acid, maleic acid, and gamma-valerolactone (GVL) were used to extract lignin during biomass pretreatment [14]. It has been considered as an important approach for clean utilization of biomass, because these organic solvents could be recycled due to their low boiling points. Nevertheless, high cost, flammability, and high operating temperatures (150–235 °C) are the challenges for their industrial applications in biomass valorization [7]. Ionic liquids (ILs) have been employed in biomass fractionation due to their some excellent characteristics, such as good thermal stability, low volatility, and recyclability [15]. The removal of lignin reached up to 50% enabling a preservation of high β-O-4′ bonds content [16]. In addition, Deep Eutectic Solvent (DES), a eutectic mixture with a hydrogen bond donor (HBD) and a hydrogen bond acceptor (HBA), enhanced the biomass biorefinery to a greener and more sustainable industry because of their notable advantages, such as nontoxicity, biodegradability, low volatility, and biocompatibility [17]. Under relatively mild conditions (120–140 °C), the lignin extraction yield exceeded 90%, which was beneficial for the efficient separation of biomass components. However, the high cost of the innovative solvents (ILs and DES) and high operation temperature of removing lignin always limit their large-scale industrial application in the lignocellulosic biomass biorefinery [18, 19]. Hence, it is urgent to search an approach for extracting high-quality lignin under a mild condition.

In previous studies, we found that *p*-toluenesulfonic acid (*p*-TsOH) aqueous solution could dissolve out hemicelluloses and lignin selectively and obtain cellulose under mild conditions (80–90 °C) [20, 21]. Besides, the acid hydrotrope can be recycled using rotary evaporation technique. For example, Ji et al. used the acid hydrotrope to pretreat *hybrid poplar* wood material at 80 °C for 20 min with a lignin-first strategy not only resulting in a high remove rate of lignin (> 90%), but also obtaining a lignin with a β-O-4′ linkage content of 60% above [22].

*Boehmeria nivea* stalk is one of the major agricultural wastes. More than 90% of the world’s *Boehmeria nivea* production is produced in China annually. Its typical disposal method is to either discard or burn for energy requirements, which not only caused a huge waste of biomass resources but also led to air pollution. Thus, it is essential to explore an efficient and mild pretreated strategy for *Boehmeria nivea* stalk to produce high value-added products.

In this study, the pretreatment of *Boehmeria nivea* stalk was carried out employing a recyclable acid hydrotrope under mild conditions (80 °C and 20 min). After pretreatment, the obtained liquid phase mainly contains dissolved lignin and hemicelluloses hydrolysates. Subsequently, a certain amount of deionized water (DI) was added to precipitate lignin. The gained lignin was converted into phenolic monomers though hydrodeoxygenation process. Under the catalysis of *p*-TsOH, the monosaccharides (mainly xylose) in spent acid liquor were degraded into furfural successfully. The obtained cellulose-rich solid was converted to fibers directly via ultrasonic treatment for 10 s, the fibers were further utilized to prepare handsheets by mixing with softwood pulp. The goal of this paper is to achieve the comprehensive utilization of *Boehmeria nivea* stalk. The novelty of this study is to provide a strategy for *Boehmeria nivea* stalk valorization using a recyclable acid hydrotrope under mild conditions. Therefore, this study is of great significance to the bio-refinery of agricultural and forestry waste biomass.

## Results and discussion

### *Boehmeria nivea* stalk pretreatment

**Fig. 1:**
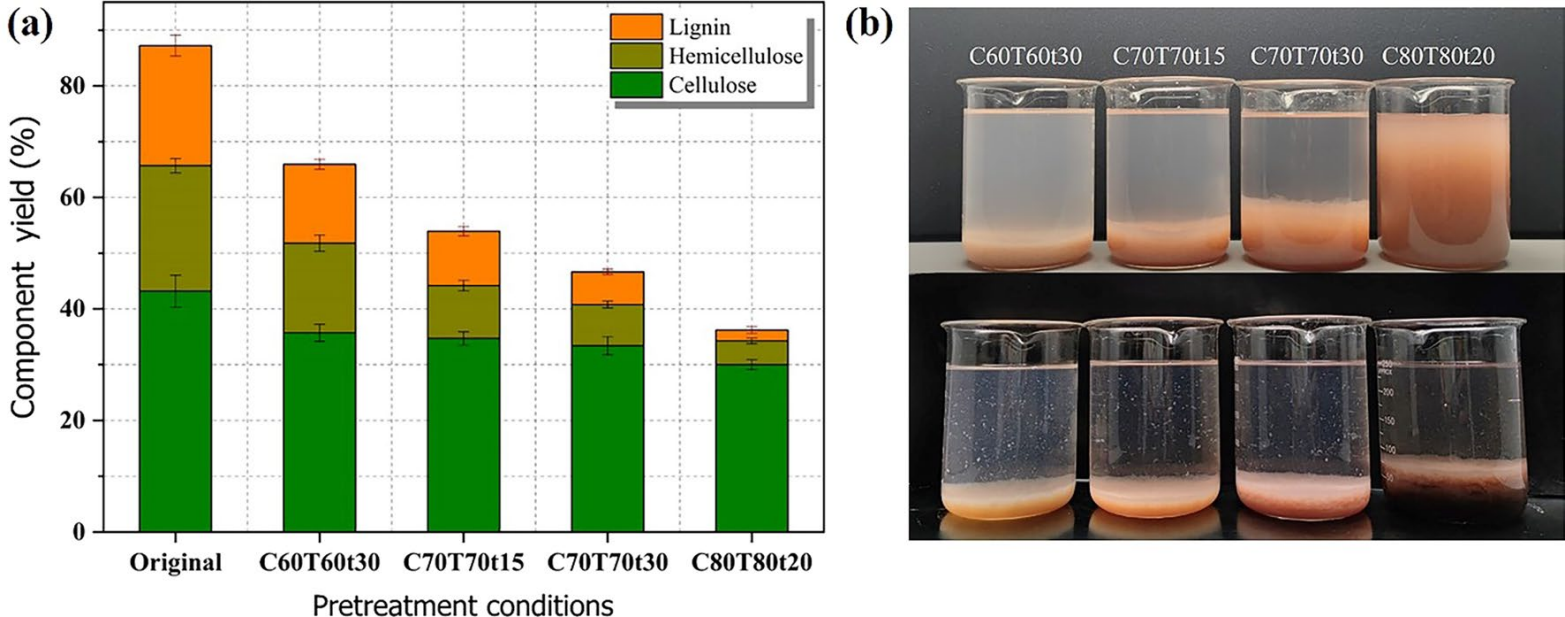
**a** The changes in three components yield for pretreated substrates under different pretreated conditions (Cx, Tx, and tx stands for the acid concentration, pretreatment temperature and pretreatment time, respectively); **b** the produced pulps (up) and extracted lignin (below) under different pretreated conditions

After pretreating under different conditions, the changes in three components of *Boehmeria nivea* stalk were displayein Fig. 1a. The main chemical components and solid yield of different pretreated samples are listed in Additional file 1: Table S1. Compared with original stalk (21.10% of hemicelluloses and 19.88% of lignin), the most of hemicelluloses and lignin were removed owing to the selective dissolution of acid hydrotrope. For instance, the obtained solid substrate after pretreatment with the condition of C70T70t30 only contained 7.39% of original hemicelluloses and 5.86% of original lignin. What’s more, increasing the pretreatment severity (temperature and time) facilitated the dissolution of two components. When the pretreatment conditions were changed from C70T70t30 to C80T80t20, hemicelluloses and lignin were removed markedly. In cell wall, the natural adhesive is attributed to the presence of lignin. After removing the extensive interactions between polysaccharides and lignin, the fibers in pretreated substrate were dispersed in deionized water to obtain pulp (Fig. 1b) by ultrasonic treatment for 10 s. Subsequently, the prepared pulp could be made use of papermaking to reduce the cost of production by blending it with commercial pulp. As shown in Fig. 1b, the fibers of C80T80t20 were completely released from the pretreated matrix in water forming pulp. Therefore, the pretreatment condition of C80T80t20 was selected to be an optimal pretreatment condition. The structural characteristics of collected lignin under various pretreatment conditions were explored via TGA, FTIR and 2D-HSQC-NMR spectroscopy, respectively. *P*-TsOH was easily recovered through rotary evaporation technique. The recovery yield reached 93.75%. Additional file 1: Fig. S1 shows the digital photo of the recycled *p*-TsOH.

### Fiber quality analysis and papermaking

The physical characteristics of fibers obtained under different pretreatment conditions were analyzed on a fiber quality analyzer as shown in Table 1. Due to poor delignification performance under C60T60t30 (28.87%), the fibers failed to be released from the pretreated matrix after 10 s ultrasonic treatment. Their physical form (Fig. 1b) cannot meet the test requirements of FQA. Therefore, the corresponding FQA results cannot be shown in Table 1. With increase of pretreatment severity from C70T70t15 to C80T80t20, the lengths of obtained fibers were reduced obviously. The phenomenon indicated that the fibers were hydrolyzed by cleaving the β-1, 4-glycosidic bonds in the presence of *p*-TsOH. Similarly, the widths of fibers decreased from 47.90 to 35.20 um, which was attributed to the further dissociation of fibers from fibrils (Additional file 1: Fig. S2). Besides, the coarseness of gained fibers was decreased with the increase of pretreatment severity (Table 1), which not only enhanced the fibers specific surface area but also improved the binding force between fibers. It was beneficial for increasing the tensile strength of handsheets. Therefore, the fiber substrates obtained under the pretreatment condition of C80T80t20 were finally selected for subsequent papermaking. The handsheets were produced via mixing gained pulp with commercial softwood pulp in various proportions through traditional papermaking technology.

**Table 1:** Quality analysis of fibers obtained from different pretreatment conditions

Sample	Coarseness (mg/m)	Kinked fibers (%)	Width (µm)
C70T70t15	1.95	5.15	47.90
C70T70t30	1.87	6.45	43.75
C80T80t20	0.06	11.95	35.20

**Fig. 2:**
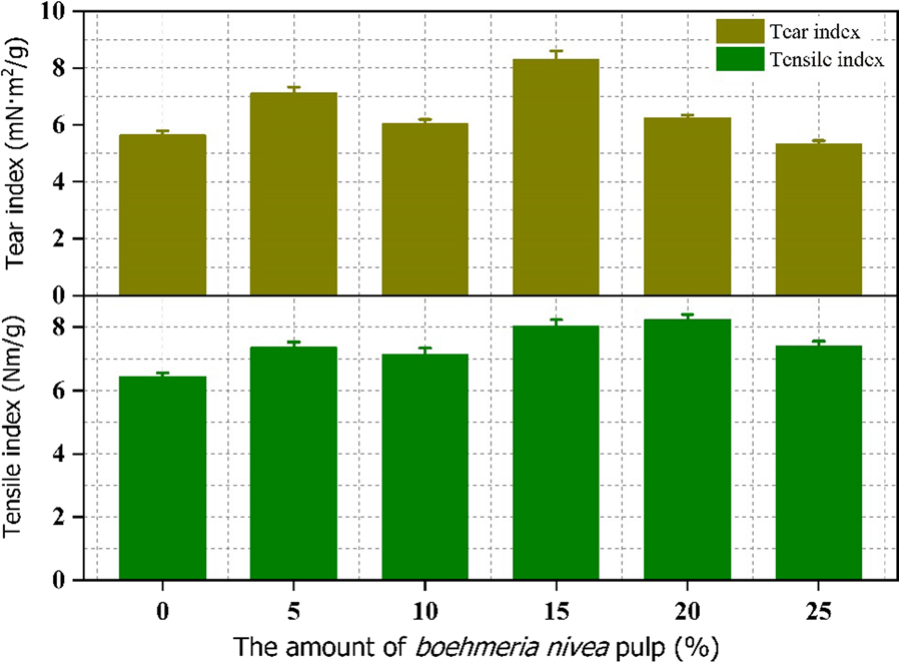
The physical strength measurements of handsheets containing different proportions of the *Boehmeria nivea* pulp and commercial softwood pulp

**Fig. 3:**
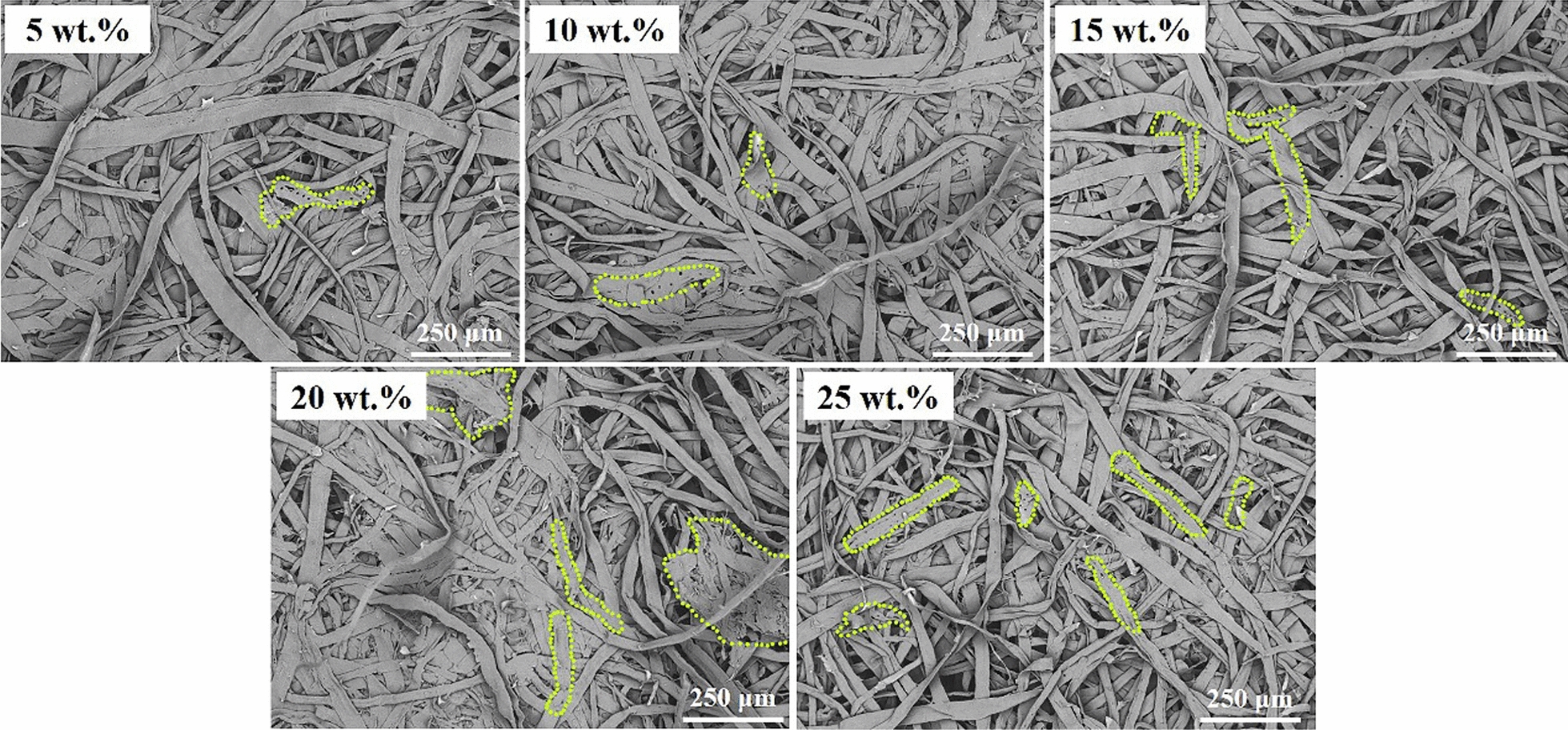
The SEM images of produced handsheets via blending different amounts of *Boehmeria nivea* pulp and softwood pulp (the pretreated fibers were marked with fluorescence)

With a basis weight of 100 g/m^2^, the produced pulp and softwood pulp were mixed with increasing proportions (0%, 5%, 10%, 15%, 20%, and 25%, wt%) to prepare handsheets according to a traditional paper fabrication approach. Figure 2 displays the tear and tensile strengths of the handsheets. As the content of produced pulp increased to 15%, the tear and tensile strengths reached up to 8.31 mN m^2^/g and 8.03 Nm/g, respectively, which was higher than that of pure softwood pulp (5.64 mN m^2^/g and 6.44 Nm/g). One of the possible explanations was that the produced short fibers from *Boehmeria nivea* stalk filled the gap between commercial pulp fibers increasing the binding sites between fibers (Fig. 3). In addition, the formed beating effect of obtained pulp by the ultrasonic treatment led the strengthening of fibrillation and increased the specific surface area, which were beneficial for exposing hydroxyl groups. Therefore, the interactions of intermolecular hydrogen bonds between the hydroxyl functional groups (–OH) of fibers were enhanced. The tear and tensile strengths of the prepared papers was improved. Increasing the proportion of the produced pulp to 25% inevitably caused reduction in the handsheets strength because of the decrease of long fiber (softwood pulp) content (Fig. 3). Therefore, blending the pretreated fibers pulp with softwood pulp for paper production could markedly decrease the cost of industrial papermaking. In a summary, reducing the consumption of commercial pulp and effectively employing the agricultural waste were achieved simultaneously adding *Boehmeria nivea* stalk pulp.

### Furfural production

**Fig. 4:**
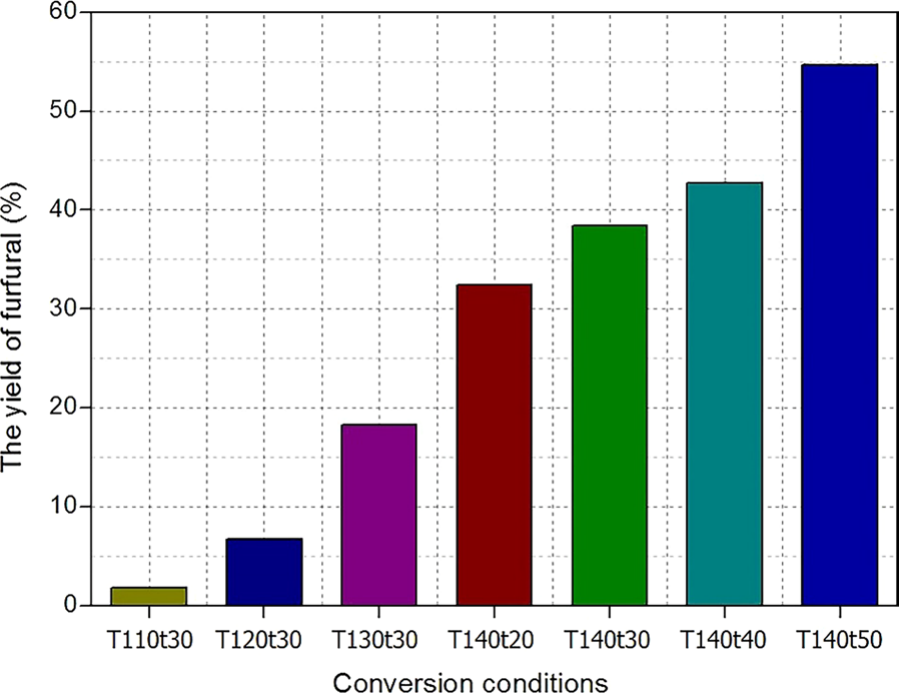
The yields of produced furfural at various reaction conditions (Tx and tx stands for reaction temperature and time, respectively)

Furfural, one of the twelve most promising molecules derived from biomass, has been identified as a top platform chemical by the US Department of Energy (DOE) [23]. After diluting the pretreatment solution with deionized water to precipitate lignin, the acid spent liquor mainly containing *p*-TsOH molecules and xylose was converted to furfural via an acid dehydration reaction [24]. The yields of furfural at various reaction conditions are shown in Fig. 4. Obviously, a high reaction severity facilitated furfural production. A maximum yield (54.67%) was obtained at T140t50 in this work. Conventional biomass conversation to furfural using acid catalysis is accompanied by the formation of black, resinous loss products that were labelled as humins by some researchers [24–26]. It was attributed to the side reactions of furfural with itself (furfural condensation) and the reactions between furfural and lignin molecules. When lignin was removed by antisolvent precipitation, this side reaction can be avoided to a certain extent. The yield of furfural, therefore, can be improved. By the furfural production, the hydrolysates of hemicelluloses were utilized. It provided a strategy of efficient utilization of hemicelluloses during the procedure of *Boehmeria nivea* stalks valorization.

### Lignin characterization and depolymerization

**Fig. 5:**
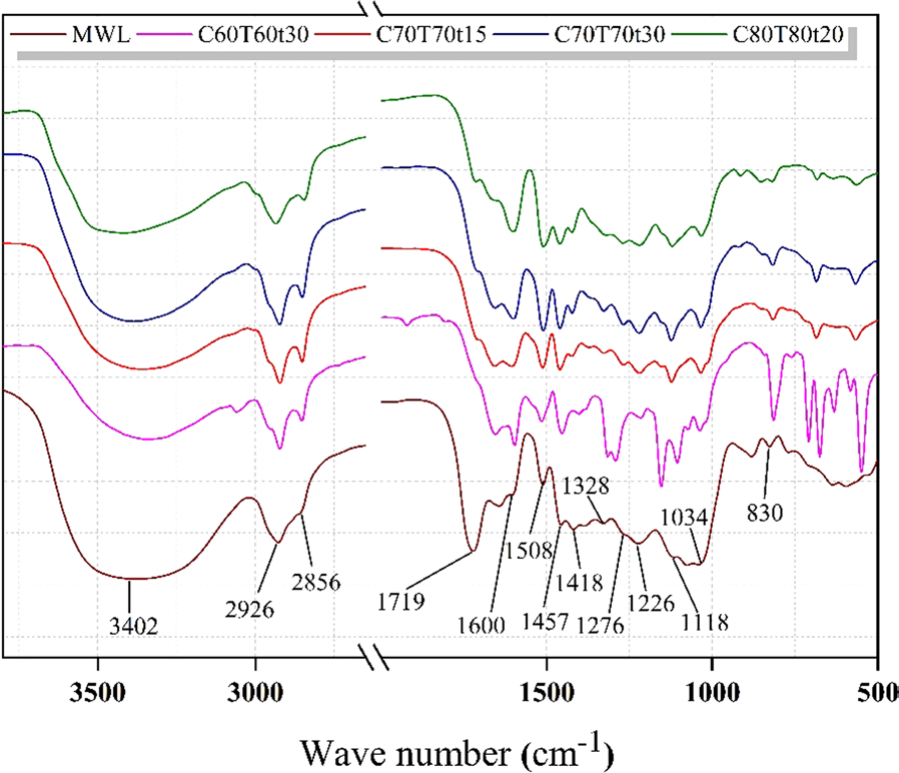
The FTIR spectra of the removed lignin from *Boehmeria nivea* stalk under various pretreatment conditions

As shown in Fig. 5, the structural properties of extracted lignin from different pretreatment conditions were analyzed through FTIR spectra. According to previous publications [27, 28], all the signals are listed in Additional file 1: Table S2. The stretching vibration of O–H was found at 3402 cm^−1^; The absorption peak at 2926 cm^−1^ and 2856 cm^−1^ was assigned to the C–H asymmetric vibrations of methyl (–CH_3_) and the C–H symmetric vibrations of methylene (–CH_2_–) [22], respectively. At the same time, several characteristic peaks of lignin also are observed in Fig. 5, such as the stretching vibration peaks of carboxyl C=O (1719 cm^−1^) and aromatic ring skeletal (1600 cm^−1^, 1508 cm^−1^ and 1418 cm^−1^) [29], the vibration peaks of benzene ring (1457 cm^−1^), and C=O bonds of guaiacyl (G) units (1118 cm^−1^), and the breathing vibration peaks of syringyl (S) (1328 cm^−1^) and condensed guaiacyl (G) (1276 cm^−1^). Besides, the peak at 1226 cm^−1^ was attributed to the stretching vibration of C–C and C=O groups [30], the peaks at 1034 cm^−1^ and 830 cm^−1^ were assigned to the aromatic C–H in-plane deformation vibrations and the C–H out-of-plane stretching vibration, respectively [31]. In summary, the pretreated lignin remained similar structure with that of natural lignin. Therefore, the collected lignin had a high-value application for multiple approaches.

**Fig. 6:**
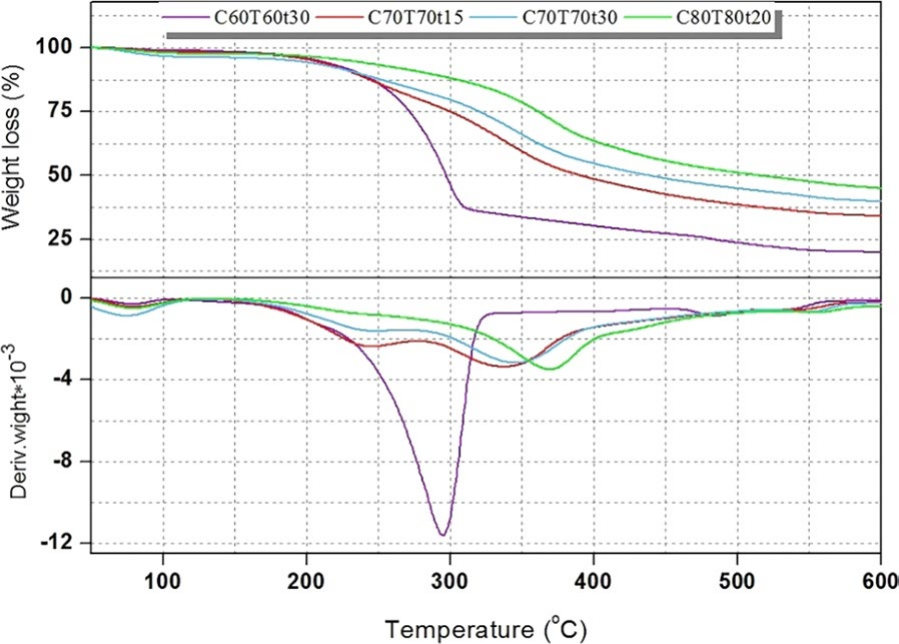
The TG and DTG curves of the obtained lignin under different conditions

The thermal stability of obtained lignin is shown in Fig. 6. The structural properties and content of functional groups of lignin samples effected their thermal stabilities. The change in weight loss of lignin samples was not obvious owing to volatilization of water molecules at the temperature range of 50–200 °C. Some weak chemical bonds, for example, the β-O-4′ linkages, also were broken during this stage. In the next temperature region (200–400 °C), almost all functional groups of lignin samples, such as aryl ether bonds, aliphatic hydroxyl groups, benzene rings, and C–C bonds were broken forming volatile substances. It can be seen from Fig. 6, after increasing the pretreatment conditions, the weight loss rate of lignin gained from *Boehmeria nivea* stalk under different conditions as the pretreatment severity increased. For example, the maximum degradation temperature of lignin under the pretreatment condition of C80T80t20 was 370 °C, which was higher than that of the lignin from C60T60t30. The results revealed the lignin was polymerized and formed stronger functional groups [22]. When the temperature exceeded 400 °C, the depletion of oxygen in lignin resulted in a low weigh loss. At the end of pyrolysis process, the lignin samples were transformed biocarbon.

The chemical structural features of extracted lignin (EL) under the pretreatment condition of C80T80t20 were analyzed by 2D HSQC NMR spectroscopy. For a comparison, a natural lignin (milled wood lignin, MWL) was prepared. Based on previous publications [32, 33], the main cross-signals are illustrated in Additional file 1: Table S3. In the benzene ring region (δ_C_/δ_H_ 100.0–136.0/6.30–8.00 ppm), the typical cross-peaks of syringyl (S) and guaiacyl (G) unites from lignin were detected clearly. For example, an obvious signal at δ_C_/δ_H_ 104.0/6.70 ppm was attributed to C_2,6_–H_2,6_ in S units. The cross-signals of G2, G5, and G6 were identified at δ_C_/δ_H_ 111.1/6.99 ppm (C_2_–H_2_, G unit), δ_C_/δ_H_ 114.5/6.70 ppm (C_5_–H_5_, G unit), and δ_C_/δ_H_ 118.9/6.79 ppm (C_6_–H_6_, G unit), respectively. In addition, the cross-peaks at δ_C_/δ_H_ 130.1/7.5 ppm and δ_C_/δ_H_ 131.6/7.6 ppm were assigned to C_2,6_–H_2,6_ in *p*-coumaric acid (PCA) unit and C_2,6_–H_2,6_ in PB unit, respectively. Compared with natural lignin, the typical cross-signals of lignin were found in the aromatic region of spectra after pretreatment.

**Fig. 7:**
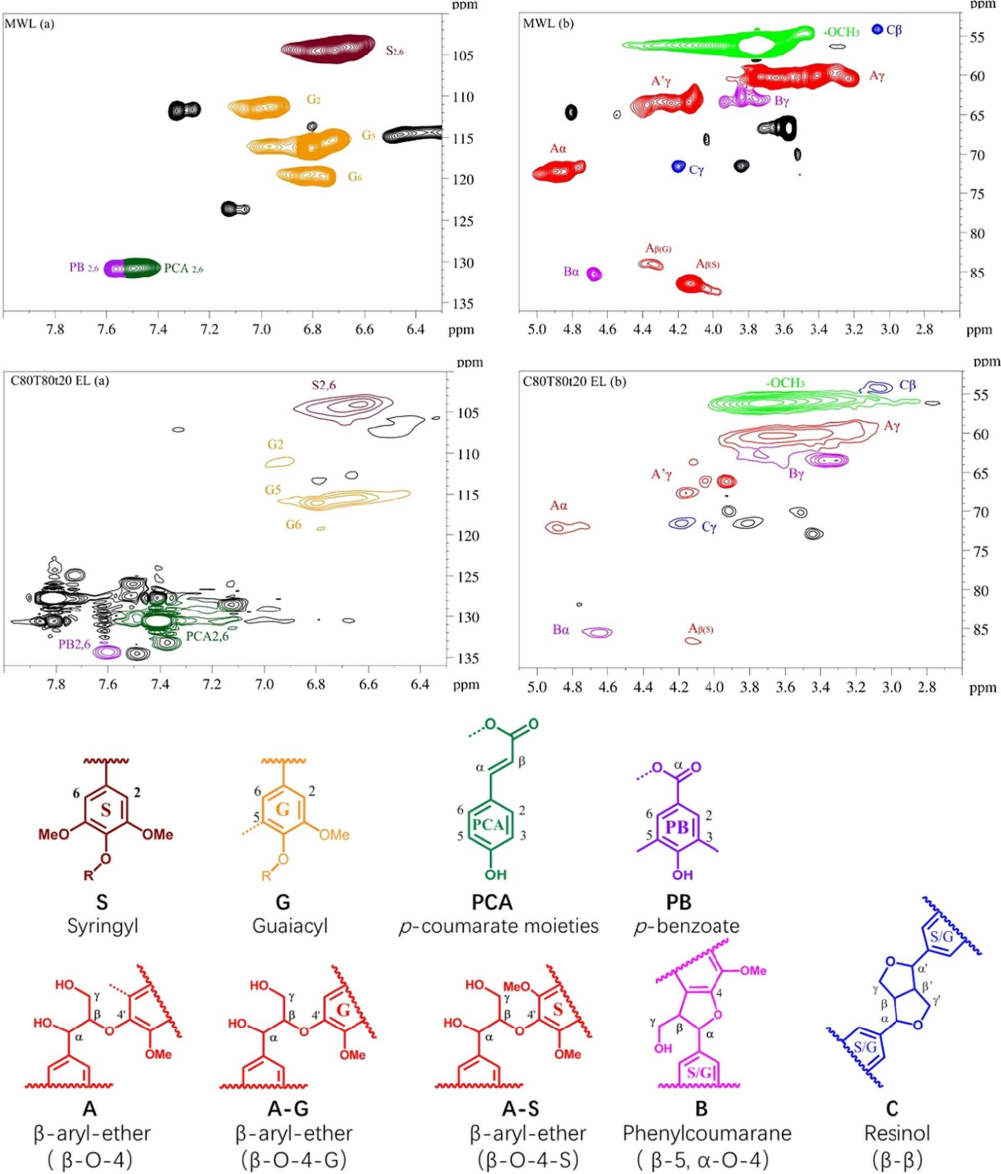
The 2D HSQC NMR spectrum and main substructures of MWL (up) and EL (below)

In the side-chain region (δ_C_/δ_H_ 52.0–90.0/2.60–5.10 ppm), typical cross-signals of interunit linkages of lignin are observed in Fig. 7. For natural lignin, the cross-peaks of methoxy groups (–OCH_3_) were observed at δ_C_/δ_H_ 55.6/3.73 ppm. Besides, other characterized signals, such as β-aryl-ether (β-O-4′, A), resinol (β-5′, B), and phenylcoumaran (β–β′, C), were also recorded in spectra. For instance, the cross-signals of β-O-4′ substructure (A unit) were detected at δ_C_/δ_H_ 71.90/4.84 ppm (C_α_-H_α_), δ_C_/δ_H_ 59.4/3.70 ppm (C_γ_-H_γ_), and δ_C_/δ_H_ 83.9–85.6/4.11–4.33 ppm (C_β_-H_β_) [34], respectively. Moreover, the β-O-4′ substructure (A′ unit) was found by the signal at δ_C_/δ_H_ 64.6/4.21 ppm (C_γ_-H_γ_). The β–β′ resinol substructures (B unit) were found according to the cross-peaks at δ_C_/δ_H_ 63.4/3.88 ppm (C_γ_-H_γ_) and δ_C_/δ_H_ 84.9/4.64 ppm (C_α_-H_α_). In addition, the signal for C_β_-H_β_ and C_γ_-H_γ_ correlations in β-5′ phenylcoumaran substructures (C unit) at δ_C_/δ_H_ 53.5/3.11 ppm and δ_C_/δ_H_ 71.2/4.21 ppm were observed in the spectra [35], respectively. For the extracted lignin, the partial strength of cross-peaks was reduced, such as β-O-4′ substructure (A′ unit) and phenylcoumaran substructures (β-5′), which may be attributed to the cleavage of β-O-4 bonds in molecules of lignin. At same time, the quantification of the major inter-unit linkages of the collected lignin is listed in Additional file 1: Table S4. The content of β-O-4′, β-5′, and β–β′ linkages in extracted lignin was 53.04%, 38.66%, and 8.3%, respectively. The extracted lignin under the condition of C80T80t20 remained similar structural properties to that of natural lignin. The results revealed that the extracted lignin from *Boehmeria nivea* stalk under the pretreatment condition of C80T80t20 had a great potential application value.

**Fig. 8:**
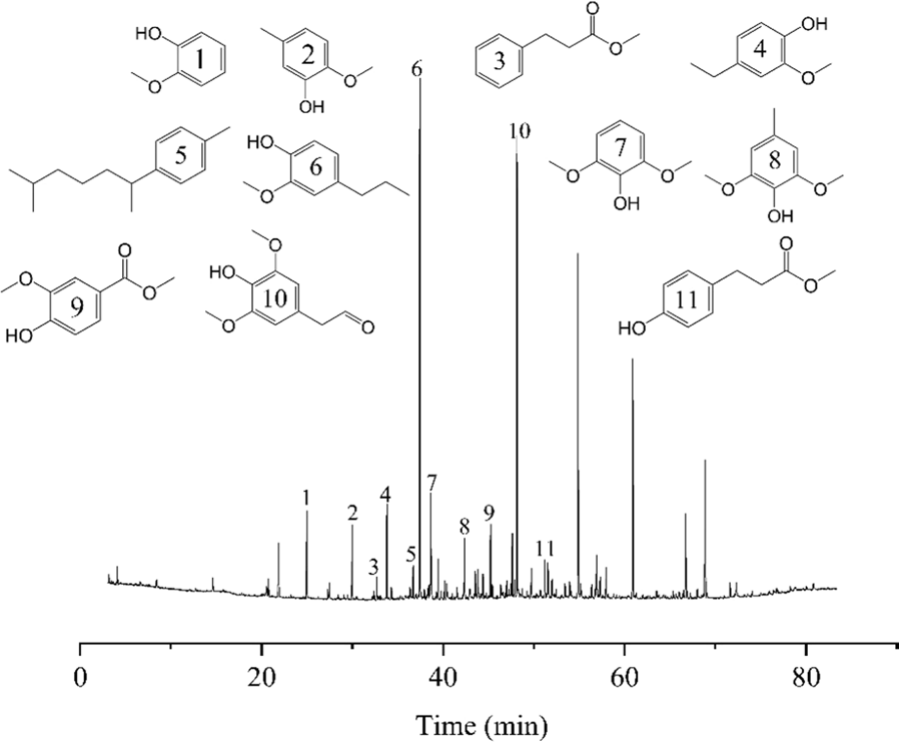
The GC–MS analysis of the bio-oil products

The reductive catalytic depolymerization of the collected lignin after pretreatment (C80T80t20) was carried out employing commercial Ru/C as catalyst in the presence of H_2_ and CH_3_OH to demonstrate its utility. After hydrogenated depolymerization, the lignin bio-oil was obtained with a high yield of 65.3%. The main produced lignin aromatics were analyzed using GC–MS as shown in Fig. 8. This indicated that the acid hydrotrope pretreatment gave rise to a low condensation degree of the removed lignin from *Boehmeria nivea* stalk. In the pretreatment process, the existence of high content of β-O-4′ linkages in lignin were beneficial to its further catalytic depolymerization. At the end of hydrogenated depolymerization, these generated lignin phenolic monomers (Fig. 8) could be utilized as diesel fuel additives to enhance the combustibility in plateau areas [36]. In addition, after further hydrogenation process, the above-mentioned phenolic monomers were directly converted to the key aviation fuel component, liquid cycloalkane [37]. In short, the collected lignin has a huge development potential in the field of producing biofuel additives.

## Conclusion

In this study, a recyclable acid hydrotrope was used for the pretreatment of *Boehmeria nivea* stalk to selectively remove hemicelluloses and lignin. Under a mild condition (C80T80t20), 90.24% of lignin and 79.86% of hemicelluloses were selectively extracted. After 10 s ultrasonic treatment, the pretreated substrates formed fibers pulp. When adding the pulp (15 wt%) in softwood pulp, the produced handsheets showed higher tear strength (8.31 mN m^2^/g) and tensile strength (8.03 Nm/g) than that of pure softwood pulp. The hydrolysates of hemicelluloses were transformed into furfural with 54.67% yield. When the extracted lignin was hydrogenated to phenolic monomers the yield of bio-oil reached 65.3%. In summary, the agricultural waste, *Boehmeria nivea* stalks, were valorized to pulp, furfural, and phenolic monomers, reaching a comprehensive utilization of lignocellulose biomass.

## Materials and methods

### Materials

*Boehmeria nivea* stalks were smashed into particles with a diameter of 0.45–0.3 mm; *p*-TsOH was purchased from Aladdin Reagent Co., Ltd (Shanghai, China); H_2_SO_4_ with a purity of 95–98% (wt%) was bought from Zouping County Tianlu Chemical Co., Ltd (Binzhou, China); All chemical reagents used were of analytical grade.

### *Boehmeria nivea* stalk pretreatment

Dry *Boehmeria nivea* stalk powder of 3 g was added in *p*-TsOH solution of 50 ml with different concentrations (60%, 70%, and 80%, wt%). Subsequently, the mixture was heated at given times (15 min, 20 min, and 30 min) for set temperatures (60 °C, 70 °C, and 80 °C). After each reaction of pretreatment, the solid phase of fiber-rich and liquid phase was separated using a filter paper (Shanghai Jinpan Biotechnology Co., Ltd., Shanghai, China) with a diameter of 15 cm. After washing with deionized water and 10 s ultrasonic treatment, the solid was completely dissociated to form pulp. Then, the collected pulp was mixed with softwood pulp to prepare handsheets. Component analysis of the produced pulp was performed on a high-performance liquid chromatography (HPLC, Ultimate 3000, Thermo Scientific) system equipped with a separating column (Aminex HPX-87H, Bio-Rad, CA, United States) and a refractive index detector (RID-20A, Shimadzu, Japan) in accordance with a standard method from National Renewable Energy Laboratory (NREL) [38].

### Fiber quality analysis and papermaking

For fiber quality analysis (FQA), the obtained fibers from *Boehmeria nivea* stalk were dispersed absolutely in fiber standard dissociator (Guangdong Fuaibo Fiber Technology Research Co., Ltd., China) and subsequently diluted with deionized water. Finally, the quality analysis of the fibers was proceeded on a L&W Fiber tester Plus (ABB AB/Lorentzen & Wettre, Sweden).

Based on the standard of SCAN-C 26:76, the various amounts of the *Boehmeria nivea* pulp (0%, 5%, 10%, 15%, 20%, and 25%, wt%) and commercial pulp were blended for papermaking with a basis weight of 100 g/m^2^. the tensile strength of the produced handsheets was detected on an Auto Tensile Tester (066, ABB AB/Lorentzen & Wettre, Sweden) according to TAPPI T 494 om-81 standard. Also, the tear strength was performed on a tearing tester (009, ABB AB/Lorentzen & Wettre, Sweden) based on the TAPPI T414 om-88 standard. Besides, the morphology of produced handsheets was observed using scanning electron microscope (SEM, TM4000Plus, Japan Hitachi Nake high-tech enterprise, Japan) at 250 μm.

### Furfural production

The xylose and *p*-TsOH molecules mainly existed in the filtered liquid phase after pretreatment. At the end of diluting the above solution using deionized water to obtain lignin, the acid spent liquor was transferred to stainless steel reactor to prepare furfural at given temperatures (110 °C, 120 °C, 130 °C, and 140 °C) for set times (20 min, 30 min, 40 min, and 50 min). After cooling to room temperature and subsequent filtering with disposable syringe filters (Tianjin Keyilong Experimental Equipment Co., Ltd, Tianjin, China), the content of generated furfural was measured at 210 nm using a HPLC system equipped with an 87H chromatography column and UV–vis detector. Subsequently, the produced furfural yield was calculated by the following equation:

$${\text{Yield}}\,\left( \% \right) = \frac{{ m_{{\text{furfural}}} }}{{ \begin{array}{*{20}c} {m_{{\text{hemicelluloses}}} /0.88 \times 0.64 } \\ \end{array} }} \times 100\%$$

where *m*_furfural_ is the weight of produced furfural; *m*_hemicelluloses_ is the weight of removed hemicelluloses from raw material under the condition of C80T80t20; 0.88 and 0.64 is conversion coefficient of hemicelluloses to xylose and xylose to furfural, respectively.

### Lignin characterization and depolymerization

For comparison, the milled wood lignin (MWL) was obtained from *Boehmeria nivea* stalk: 10 g of raw materials was milled 4 h by planetary ball mill at 4000 rpm. Under the function of cellulase (147 FPU/ml, Cellic^®^ CTec2), the Enzymatic hydrolysis reaction was conducted using a shaking table under conditions of 50 °C, 180 rpm and 24 h. After centrifugal dewatering and freeze drying, the residual solid was added to a mixed solution of dioxane and water with a volume ratio of 94 to 4, and the MWL was extracted on a shaker (35 °C, 180 rpm, 24 h) in the dark.

The FTIR measurement of the extracted lignin (EL) was performed on an infrared spectrometer (NEXUS 670, Thermo Nicolet). The scanning wavelength range and resolution were 500–4000 cm^−1^ and 4 cm^−1^, respectively.

The TGA measurement was carried out on a thermogravimetric analyzer (NETZSCH, Germany) under nitrogen atmosphere. The test temperature was set at 35–800 °C and the heating rate was given at 10 °C/min.

For 2D-HSQC NMR analysis, after thoroughly blending the DMSO-*d*6 of 0.6 ml and dry lignin sample of 40 mg, the solution was immediately transferred to an NMR tube. Subsequently, the solution was scanned on a 400 MHz spectrometer (BRUKER, Germany) at room temperature.

The depolymerization of the extracted lignin was performed in high pressure reactor containing hydrogen, lignin of 0.3 g, Ru/C catalyst of 0.3 g, and methanol of 20 ml. The initial hydrogen pressure was 1 Mpa. The hydrodeoxygenation reaction was carried out at a temperature of 230 °C for 15 h. The working pressure was 8 MPa. First of all, the reactor was cooled to room temperature after the reaction. The liquid and solid phases were separated by filtration. Then, the former was extracted completely adding n-hexane. Finally, the analysis of partial liquid was conducted on a GC–MS system (QP2020, Shimadzu) fitting out a chromatographic column of HP-5, the remaining liquid was collected and concentrated via rotary evaporation (RE-52AA, Shanghai Yarong Biochemistry Instrumentfactory). Subsequently, the yield of depolymerization products was calculated by the following equation:

$${\text{Yield}}\,\left( \% \right) = \frac{{m_{\text{bio - oil}} }}{{m_{{\text{lignin}}} }} \times 100\%$$

where *m*_bio-oil_ is the weight of bio-oil obtained after lignin depolymerization and *m*_lignin_ is the original weight of lignin used.

## Supplementary Information

**Additional file 1. Table S1** The main chemical components and solid yield of different pretreated samples; **Table S2** The absorption peak and corresponding groups of FTIR spectra; **Table S3** The main cross-signals and corresponding groups of the benzene ring and side-chain; **Table S4** The quantification of the major inter-unit linkages in lignin by 2D HSQC NMR spectroscopy; **Fig. S1** The digital photo of recycled p-TsOH; **Fig. S2** The SEM image of fiber under the pretreatment condition of C80T80t20.

## Data Availability

Data will be made available on request.
