# Supplementary material for: Valorization of Boehmeria nivea stalk towards multipurpose fractionation: furfural, pulp, and phenolic monomers
Source: Biotechnol Biofuels Bioprod. 2023 Jun 12;16:99. doi: 10.1186/s13068-023-02351-x (PMC10262554; doi:10.1186/s13068-023-02351-x)
Supplement: Supplementary file 1 — Additional file 1. Table S1 The main chemical components and solid yield of different pretreated samples; Table S2 The absorption peak and corresponding groups of FTIR spectra; Table S3 The main cross-signals and corresponding groups of the benzene ring and side-chain; Table S4 The quantification of the major inter-unit linkages in lignin by 2D HSQC NMR spectroscopy; Fig. S1 The digital photo of recycled p-TsOH; Fig. S2 The SEM image of fiber under the pretreatment condition of C80T80t20. [file 13068_2023_2351_MOESM1_ESM.docx]

Supplementary materials

Valorization of *Boehmeria nivea* stalk towards Multipurpose Fractionation: Furfural, Pulp, and Phenolic monomers

Zhen Zhang ^1^, Furong Tao ^2^, Hairui Ji ^1*^

^1^ Key Laboratory of Pulp and Paper Science & Technology of Ministry of Education, State Key Laboratory of Biobased Material and Green Papermaking, Faculty of Light Industry, Qilu University of Technology (Shandong Academy of Sciences), 250353, Jinan, China.

^2^ Faculty of Chemistry and Chemical Engineering, Qilu University of Technology (Shandong Academy of Sciences), 250353, Jinan, China.

*Corresponding author: jihairui@yeah.net

Table S1 The main chemical components and solid yield of different pretreated samples

| Samples | Cellulose (%) | Hemicellulose (%) | Lignin (%) |
| --- | --- | --- | --- |
| Original | 40.58±2.87 | 21.10±1.28 | 19.88±1.88 |
| C60T60t30 | 35.69±1.53 | 16.09±1.44 | 14.14±0.90 |
| C70T70t15 | 34.70±1.19 | 9.49±0.94 | 9.72±0.82 |
| C70T70t30 | 33.40±1.62 | 7.39±0.62 | 5.86±0.47 |
| C80T80t20 | 30.02±0.90 | 4.25±0.51 | 1.94±0.61 |


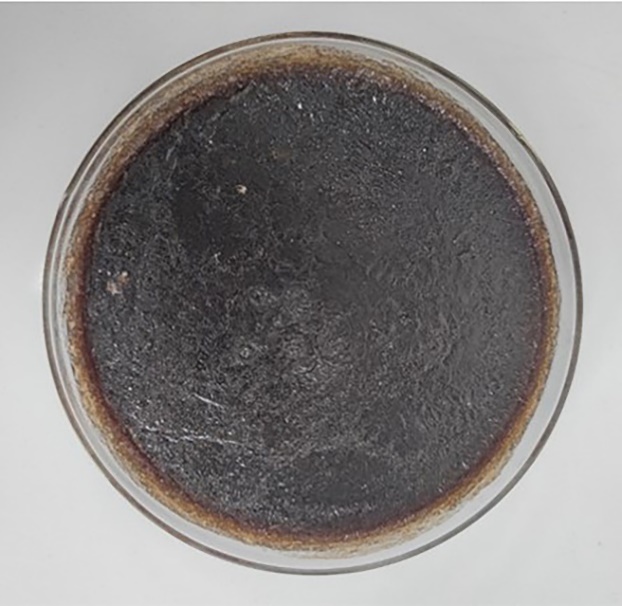


Fig.S1 The digital photo of recycled *p*-TsOH


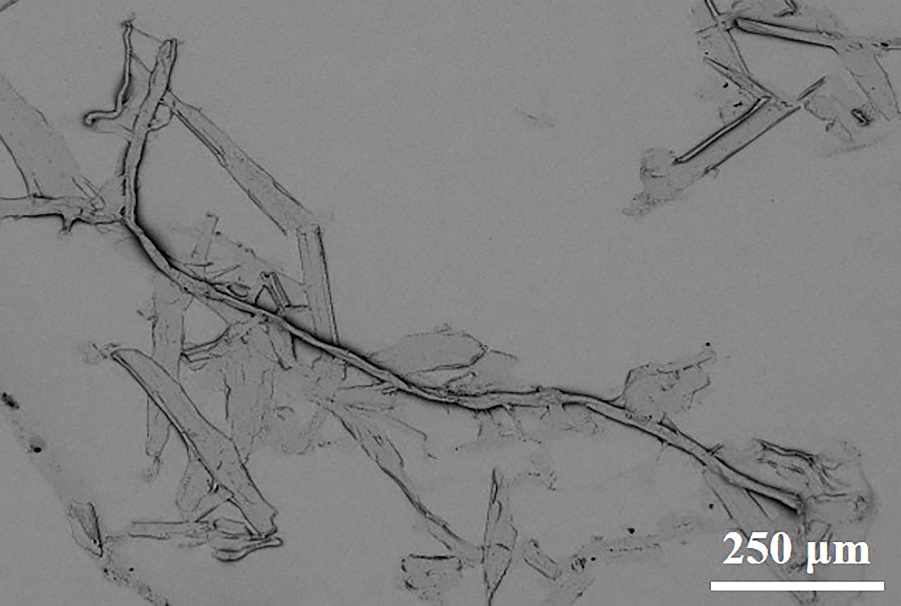


Fig.S2 The SEM image of fiber under the pretreatment condition of C80T80t20

Table S2 The absorption peak and corresponding groups of FTIR spectra

| Wave number (cm^-1^) | Assignment |
| --- | --- |
| 3402 | stretching vibration of O-H |
| 2926 | C-H asymmetric vibrations of -CH3 |
| 2856 | C-H symmetric vibrations of -CH2- |
| 1719 | stretching vibration of carboxyl C=O |
| 1600/1508/1418 | stretching vibration of aromatic ring skeletal |
| 1457 | vibration of benzene ring |
| 1328 | breathing vibration of S units |
| 1276 | breathing vibration of condensed G units |
| 1226 | stretching vibration of C-C and C=O |
| 1118 | vibration of C=O bonds of G units |
| 1034 | aromatic C-H in-plane deformation vibrations |
| 830 | C-H out-of-plane stretching vibration |

Table S3 The main cross-signals and corresponding groups of the benzene ring and side-chain

| Label | δ_C_/δ_H_ (ppm) | Assignment |
| --- | --- | --- |
| Aα | 71.90/4.84 | Cα-Hα in β-O-4′substructures (A) |
| Aγ | 59.4/3.70 | Cγ-Hγ in β-O-4′substructures (A) |
| A′γ | 64.6/4.21 | Cγ–Hγ in γ-acylated β–O–4′substructures (A′) |
| Aβ(G) | 83.9/4.33 | C_β_−H_β_ in β-O-4’ linked to G unit (A) |
| Aβ(S) | 85.6/4.11 | C_β_−H_β_ in β-O-4’ linked to S unit (A) |
| Bα | 84.9/4.64 | Cα-Hα in phenylcoumaran substructures (B) |
| Bγ | 63.4/3.88 | Cγ-Hγ in phenylcoumaran substructures (B) |
| Cβ | 53.5/3.11 | C_β_-H_β_ in β-β′resinol substructures (C) |
| Cγ | 71.2/4.21 | Cγ-Hγ in β-β′resinol substructures (C) |
| -OCH3 | 55.6/3.73 | C-H in methoxyls |
| S_2,6_ | 104.0/6.70 | C_2,6_-H_2,6_ in syringyl units (S) |
| G_2_ | 111.1/6.99 | C_2_−H_2_ in guaiacyl units (G) |
| G_5_ | 114.5/6.70 | C_5_−H_5_ in guaiacyl units (G) |
| G_6_ | 118.9/6.79 | C_6_−H_6_ in guaiacyl units (G) |
| PCA _2,6_ | 130.1/7.5 | C_2,6_-H_2,6_ in p-coumaric acid (PCA) |
| PB _2,6_ | 131.6/7.6 | C_2,6_-H_2,6_ in p-benzoate (PB) |

Table S4 The quantification of the major inter-unit linkages in lignin by 2D HSQC NMR spectroscopy

| Sample | β-O-4’ | β-5’ | β-β’ | S/G |
| --- | --- | --- | --- | --- |
| C80T80t20 | 53.04 | 38.66 | 8.30 | 7.90 |
